# Somatostatin Receptors as Molecular Targets in Human Uveal Melanoma

**DOI:** 10.3390/molecules23071535

**Published:** 2018-06-26

**Authors:** Kristof Harda, Zsuzsanna Szabo, Erzsebet Szabo, Gabor Olah, Klara Fodor, Csaba Szasz, Gabor Mehes, Andrew V. Schally, Gabor Halmos

**Affiliations:** 1 Department of Biopharmacy, University of Debrecen, 4032 Debrecen, Hungary; harda.kristof@pharm.unideb.hu (K.H.); szabo.zsuzsanna@pharm.unideb.hu (Z.S.); erzsebet.szabo@med.unideb.hu (E.S.); olah.gabor@pharm.unideb.hu (G.O.); fodor.klara@pharm.unideb.hu (K.F.); 2 Department of Pathology, University of Debrecen, 4032 Debrecen, Hungary; szasz.csaba@med.unideb.hu (C.S.); gabor.mehes@med.unideb.hu (G.M.); 3 Endocrine, Polypeptide and Cancer Institute, Veterans Affairs Medical Center, Miami, FL 33125, USA; ASchally@med.miami.edu; 4 Department of Pathology, Miller School of Medicine, University of Miami, Miami, FL 33101, USA; 5 Department of Medicine, Divisions of Hematology-Oncology and Endocrinology, Miller School of Medicine, University of Miami, Miami, FL 33101, USA; 6 Sylvester Comprehensive Cancer Center, University of Miami, Miami, FL 33136, USA

**Keywords:** human uveal melanoma, somatostatin receptors, molecular targets SSTR-1-5, cancer therapy

## Abstract

Uveal melanoma (UM) is the most common primary intraocular malignancy in adults, with an incidence of 4–5 cases per million. The prognosis of UM is very poor. In the present study, our aim was to investigate the expression of mRNA and protein for somatostatin receptor types-1, -2, -3, -4, -5 (SSTR-1–5) in human UM tissue samples and in OCM-1 and OCM-3 human UM cell lines by qRT-PCR, western blot and ligand competition assay. The mRNA for SSTR-2 showed markedly higher expression in UM tissues than SSTR-5. The presence of SSTRs was demonstrated in 70% of UM specimens using ligand competition assay and both human UM models displayed specific high affinity SSTRs. Among the five SSTRs, the mRNA investigated for SSTR-2 and SSTR-5 receptors was strongly expressed in both human UM cell lines, SSTR-5 showing the highest expression. The presence of the SSTR-2 and SSTR-5 receptor proteins was confirmed in both cell lines by western blot. In summary, the expression of somatostatin receptors in human UM specimens and in OCM-1 and OCM-3 human UM cell lines suggests that they could serve as a potential molecular target for therapy of UM using modern powerful cytotoxic SST analogs targeting SSTR-2 and SSTR-5 receptors.

## 1. Introduction

Uveal melanoma is the most common primary intraocular malignancy in adults [1], with a yearly incidence of 4–5 cases per million [2]. The intraocular uveal tract is comprised of the iris, choroid and ciliary body. The reason for malignant UM formation is unknown, but various predisposition factors have been associated with the development of this quite aggressive disease. A few personal features, such as fair complexion [3], light irides [4,5], uveal naevi [6], dysplastic naevus syndrome [7,8], oculodermal and ocular melanocytosis [9] and neurofibromatosis type 1 (NF1) [10,11], have been connected with an increased chance of UM [12]. The mortality due to UM has remained relatively unchanged, in spite of earlier detection and consequently smaller primary tumor burdens [13,14]. Approximately half of all patients with UM will eventually develop metastatic disease. It is generally supposed that the primary reason for treating UM is to avoid metastatic spreading of the tumor from the eye [13,15]. The liver is the most common site of metastases and once liver metastases are clinically apparent the prognosis becomes poor. One of the most significant predictors for UM-related death is loss of chromosome 3 [16,17,18]. The incidence of UM in whites is eight times higher than in blacks [19]. The leading predictors of survival for UM are histologic cell type, largest tumor diameter, age, gender and tumor location [20]. More unbiased classification parameters have appeared from comprehensive cytomorphometrical studies. Life expectancy of UM patients with metastatic disease depends on the rapidity of the metastatic process. In order to reduce the mortality caused by melanoma, it is essential to prevent or eliminate metastatic disease. This requires early detection and the improvement of prognostic factors [20,21]. It is crucial to increase our knowledge of the mechanism of metastasis and describe reliable progression parameters as prognostic markers in primary UM [20]. These facts inspire a constant search for new concepts to improve quality of life and extend survival of patients. For patients with UM there is no effective therapy if metastases have developed. Despite several therapeutic strategies and successful eradication of the ocular tumor, metastatic disease is almost always fatal [21].

Uveal melanoma consists of a well-defined population of melanocytes, from which uveal malignant melanoma originates. As melanocytes originate in the neuroectodermal-neural crest, it is theorized that melanoma shows characteristics similar to neuroendocrine tumors. Somatostatin was initially discovered as a hypothalamic neurohormone. It is widely distributed in the central and peripherial nervous system and has also been found in the endocrine pancreas, gut, thyroid, adrenal glands, and kidneys [22,23,24].

Somatostatin receptors (SSTRs) have been shown to be expressed in several different types of cancer, including uveal melanoma [22,24,25,26]. There are two different forms of bioactive peptides which are produced in mammals. Somatostatin-14 is a cyclic peptide including 14 amino acids, and somatostatin-28 consists of 28 amino acids. Somatostatin (SST) acts mainly as an inhibitory factor in cell proliferation and hormone secretion with endocrine, paracrine and autocrine activities [23]. At least five subtypes of SSTRs have been characterized in humans (1, 2A, 2B, 3, 4, and 5), and SSTR-2 and SSTR-5 are the ones mostly studied in human cancers including primary UM. The genes for these subtypes are located on different chromosomes [22,27]. SSTR-2 otherwise splices to generate two isoforms named SSTR-2A and SSTR-2B, which deviate in their C-terminal sequences. They all bind to SST-14 and -28, but they have a slightly higher affinity to SST-14 [28].

Somatostatin could be involved in tumor growth suppression, as confirmed by the use of SST analogs to treat neuroendocrine tumors [24,29,30]. Earlier in vitro and in vivo experimental studies have already demonstrated the inhibitory effect of various SST analogs and cytotoxic SST analog AN-162 in breast cancer, lung cancer, glioblastoma, and colon carcinoma, suggesting the potential application of modern powerful cytotoxic SST analogs in patients suffering from malignant tumors [23,24,26,30,31,32,33,34,35]. Even if each tumor expresses more than one subtype of SSTRs, SSTR-2 is the most regularly observed. The biological effects of SST are mediated by specific G protein-coupled plasma membrane receptors, which are placed in specific target cells of the gastrointestinal [36] tract, the peripheral nervous system and various blood vessels.

As a result of the similar origin of SST and UM cells, a possible correlation or interaction can be found. A connection between eye tissue and neurohormones has already been discovered in several studies [37,38,39]. However, there is still limited information about SSTR expression and characterization in human UM. Therefore, we aimed to widen our research regarding the expression of SSTR types in human UM, in order to recognize specific membrane receptors as molecular targets for diagnostic and therapeutic purposes.

In the present study, our goal in the experimental work was to examine the expression of mRNA for somatostatin receptor types-1, -2, -3, -4 and -5 (SSTR-1–5) in human UM cell lines and tissue samples. We also aimed to study the presence and binding characteristics of SSTR protein by Western blot and ligand competition assays. An additional goal was to compare our results with clinicopathological data in order to better understand their diagnostic and therapeutic significance.

## 2. Results

### 2.1. Expression of SSTRs in Human UM Tissue Samples

In our study 46 human UM tissue samples were investigated. The clinicopathological data of patients are shown in Table 1. Twenty nine samples were obtained from men and 17 samples from women. More than half of the patients (24, 52%) were over 66 years of age, while the remaining samples were equally distributed among the different age groups.

Histopathological classification of these samples revealed that 10 of them belonged to spindle type. Seven samples were determined to be epithelioid type and 2 samples were mixed of spindle and epithelioid types. Two samples were not classified histologically. Due to the limited amount of good quality RNA isolated from UM tissue samples. 46 specimens were studied for the expression of mRNA for SSTR-2 and nine specimens for SSTR-5.

**Figure 1:**
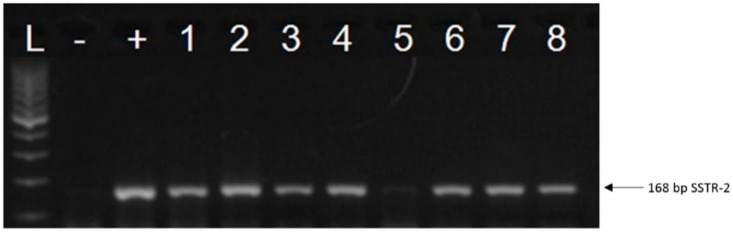
A representative figure of the expression of SSTR-2 in human uveal melanoma. L: 100 bp DNA Ladder; “−”: negative control; “+”: positive control (UM pool); 1–5: representative human uveal melanoma tissue samples; 6: CACO-2 cell line; 7: OCM-1 human uveal melanoma cell line; 8: OCM-3 human uveal melanoma cell line.

**Figure 2:**
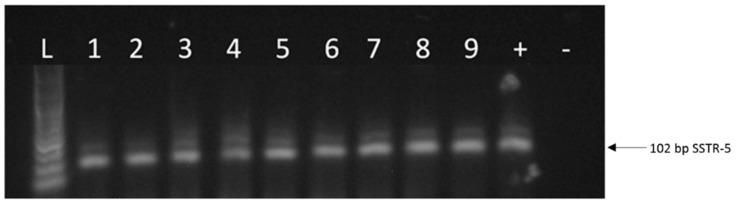
A representative figure of the expression of SSTR-5 in human uveal melanoma. L: 100 bp DNA Ladder; 1–6: representative human uveal melanoma tissue samples; 7: CACO-2 cell line; 8: OCM-1 human UM cell line; 9: OCM-3 human UM cell line. “+”: positive control (UM pool); “−”: negative control.

The expression of mRNA for SSTR-2 was detected in 30 of the 46 human UM specimens (65.2%) (Table 1, Figure 1). Nine human UM tissue samples were tested for SSTR-5 expression and we found that 66.6% (6 specimens) of these samples were positive for SSTR-5 (Table 1, Figure 2). Among the positive cases for SSTR-2, 60.0% were men and 40.0% women (Table 1). The distribution of SSTR-2-positive samples by age group, showed the following findings: age 30–45 years: 62.5%; age 46–55: 75.0%; age 56–65: 66.7%; age above 66 years: 66.7%. There was no correlation between the expression of different SSTR-types and age groups. 40% of the samples positive for SSTR-2 were spindle, 36.7% were epithelioid and 10% were mixed histological type. Four SSTR-5-positive samples were spindle (66.7%) and two specimens were epithelioid (33.3%).

The presence of SSTRs, characteristics of these SST-binding sites and specific binding of radioiodinated RC-160 to membrane receptors on human UM samples were determined using ligand competition assays. Of the 20 specimens examined, 14 (70%) showed SSTR binding (Table 1). Receptor binding affinity and concentration of SSTRs in tumor membranes were measured in displacement experiments. Analyses of the typical displacement curves of [^125^I]RC-160 by the same unlabeled peptide (RC-160) showed that the one-site model could provide the best fit. Based on these results the presence of one class of high affinity SSTR in crude membranes derived from human UM samples was indicated. The computerized non-linear curve-fitting program and the Scatchard plot analyses of the SST receptor binding data in 14 receptor positive tumor samples showed that the single class of binding sites had a mean dissociation constant (K_d_) of 7.14 nM (range 3.18–11.8 nM), with a mean maximal binding capacity (B_max_) of 604.0 fmol/mg membrane protein (range 260–1052 fmol/mg protein). Biochemical specifications and parameters crucial for characterizing specific binding sites were also defined. Thus, the binding of [^125^I]RC-160 was detected to be reversible, temperature- and time-dependent, and linear with protein concentration in the human UM specimens examined (data not shown). Competitive binding studies also demonstrated the specificity of SST binding sites using numerous peptides structurally related or unrelated to SST. The binding of radiolabeled RC-160 was displaced completely by increasing concentrations (10^−12^–10^−6^ M) of SST-14, whereas none of the structurally and functionally different and unrelated peptides analyzed, such as luteinizing hormone-releasing hormone (LHRH), growth hormone-releasing hormone (GHRH), epidermal growth factor (EGF), [Tyr^4^]bombesin, and insulin-like growth factor I inhibited the binding of radioiodinated RC-160 at concentrations as high as 1 µM (data not shown).

### 2.2. Expression of SSTRs in Human UM Cell Lines

**Figure 3:**
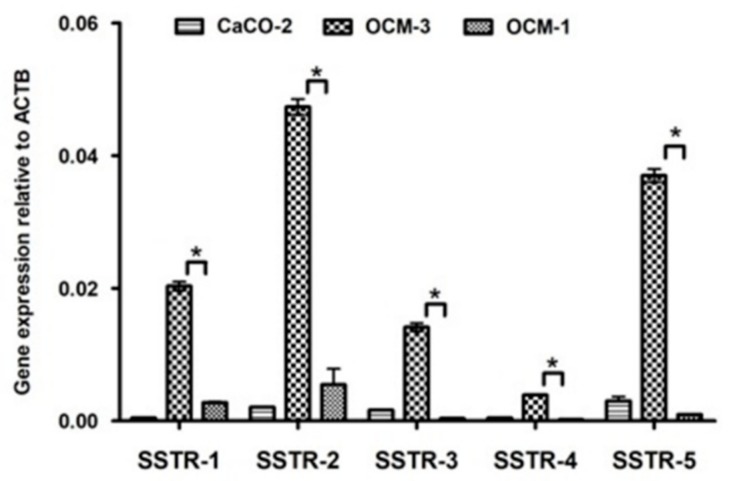
Expression of SSTR-1-5 in OCM-1, OCM-3 human uveal melanoma cell lines and in CACO-2 human Caucasian colon adenocarcinoma cell lines. qRT-PCR results show that SSTR-1, -2, -3, -4, -5 are expressed in both human UM cell lines examined. CACO-2 cell line was used as a positive control. The *p* value obtained by GraphPad Prism—one-way ANOVA test was considered to be significant (* *p* < 0.0001).

**Figure 4:**
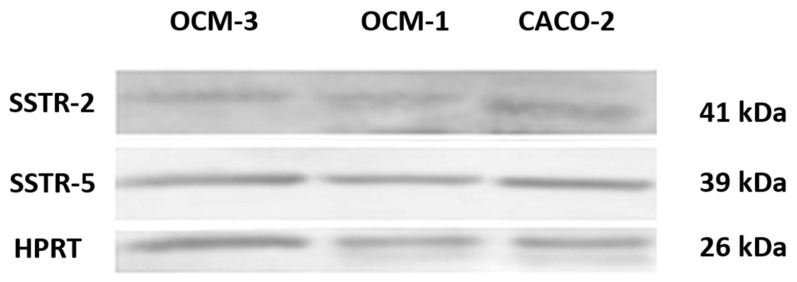
Western blot analysis of SSTR-2 and SSTR-5 protein in human cell lines.

Our RT-PCR and qRT-PCR results clearly show that SSTR-1-5 were expressed in both human UM cell lines examined. CACO-2 cell line as a positive control also showed the expression of all five SSTRs examined. The highest expression of all the investigated SSTRs was observed in OCM-3 cell line. However, in both cell lines, the expression of SSTR-2 and -5 was stronger than those of SSTR-1, -3 and -4. All five SSTRs examined displayed significantly (*p* < 0.0001) higher expression in OCM-3 UM cell line than in OCM-1 (Figure 3). Western blot analysis also confirmed the presence of SSTR-2 and SSTR-5 receptor protein in OCM-1 and OCM-3 human UM cell lines. Similarly to our findings on receptor mRNA, higher expression levels of SSTR-2 and SSTR-5 receptor protein were found in OCM-3 tumor cell line, than in OCM-1 cell line detected by western blot (Figure 4).

In cell membranes of OCM-1 and OCM-3 human UM cell lines, ligand competition studies also revealed a single class of high affinity binding sites for SST with a mean dissociation constant (K_d_) of 5.34 and 6.72 nM, respectively. The concentration of SSTRs was 433 fmol/mg membrane protein in OCM-1 cells, while OCM-3 cells showed a markedly higher receptor level (981 fmol/mg membrane protein).

Western blot analysis confirmed the presence of SSTR-2 and SSTR-5 receptor proteins in OCM-1 and OCM-3 human UM cell lines. CACO-2 human Caucasian colon adenocarcinoma cell line was used as a positive control. Anti-HPRT (1:1000 dilution) was used as a housekeeping gene. A description of experimental conditions is found in the Materials and Methods section.

## 3. Discussion

Uveal melanoma is the most frequently diagnosed primary malignant intraocular tumor in adults, and the second most common form of melanoma after cutaneous melanoma [20,40]. Over the past 50 years, despite advances in diagnosis and effective local therapies, stage-specific UM mortality rates have remained essentially unchanged and continue to be associated with significant mortality. The liver is the most common and important target of metastasis, and liver failure due to metastasis is the immediate cause of death in most patients [15,36].

Patients with UM face a gloomy prognosis, as about 45% die due to metastasis, irrespective of the fact that the tumor is most frequently diagnosed and locally cured before any signs of clinical distributed disease appear [41]. This led to the theory that micrometastases are already present early in the disease process, but they remain inactive for many years before a clinically noticeable macrometastasis develops [1].

Tumors of the ciliary body and choroid pose a severe threat to life. Treatment of primary uveal melanoma is still disputed. It is still mostly agreed that enucleation is unavoidable for many large ciliary body and choroidal melanomas. It seems justified to subject clinically relatively small, “active” melanomas to some type of treatment. Many large melanomas and most medium-sized melanomas are being treated nowadays by irradiation with or without thermotherapy, or by enucleation. It has been suggested that modifications of local treatment will not result in any remarkable improvement in survival, and that research must be directed towards treatment of metastatic disease [36].

The systemic treatment of UM is very limited. Adjuvant systemic therapy is primarily used in patients at high risk of metastasis or developed metastatic patients, but the response rates for classic chemotherapeutic agents remain between 7% and 25% [42]. Systemic chemotherapy for the treatment of UM is ineffective. In spite of new insights into the genetic and molecular background of metastatic UM, satisfactory systemic treatment approaches are currently lacking [43]. There are currently no effective systemic therapies available that could be used for the primary tumor. Despite the improvement in the diagnosis and therapy of primary UM, the number of metastatic deaths has not been significantly reduced over the past 20 to 30 years [44,45]. Therefore, innovative therapeutic methods are urgently awaited [24,25,46,47,48].

Both forms of the hypothalamic neuropeptide somatostatin (somatostatin-14 and -28), also exist in the gastrointestinal tract and their function is to inhibit the secretion of many hormones including growth hormone, insulin and gastrin, glucagon, secretin and cholecystokinin. In addition to its general endocrine effects, SST can function as an autocrine/paracrine regulator and is also present in the gastric mucosa, pancreas, and duodenum, where it suppresses exocrine secretions including gastric acid and pancreatic enzymes, respectively [49].

Because of the short plasma half-life of SST-14 (3 min), more stable and more potent synthetic SST analogs have been developed, including octreotide (Sandostatin^®^) [50], vapreotide (RC-160, Octastatin^®^), and lanreotide (Somatulin^®^) [26,51]. The plasma half-life of SST analogs is 120 min, and these peptide analogs are about 50 times more potent than SST in inhibiting growth hormone release from the pituitary. It has been well published that SST and its octapeptide analogs exert their effects through specific G-protein coupled membrane receptors [26,52]. While native SST shows similar high affinity to SSTR-1-5, the synthetic octapeptides such as octreotide, RC-160, and RC-121 bind especially to SSTR-2 and SSTR-5, present a moderate affinity to SSTR-3, and a low affinity to SSTR-1 and SSTR4 [49]. Somatostatin analogs, including RC-160, have been shown to function as effective tumor growth suppressors in experimental models of various cancers. To increase therapeutic efficacy, new analogs have been prepared which contain the cyclohexapeptide, designated SOM230 (pasireotide), which binds with high affinity to SSTR-1, -2, -3 and SSTR-5 [53]. The presence of somatostatin receptors, mainly SSTR-2 on tumors, allows the localization of certain primary tumors and their metastases using scintigraphic techniques. Radiolabeled analogs of SST, such as [^111^In-DTPA-D-Phe1]-octreotide (OctreoScan^®^) are used clinically for the localization of tumors expressing receptors for somatostatin. Targeted radiotherapy, in which somatostatin analogs are linked to numerous radionuclides such as ^68^Gallium or ^90^Yttrium, is also advancing clinically [30,54,55,56].

In this study, the expression of mRNA for SSTR-2 was studied in 46 UM specimens, and SSTR-5 in 9 UM tumor samples. Primary tumors were also tested at the protein level. In addition to detecting SSTRs, OCM-1 and OCM-3 UM cell lines were also analyzed for receptor mRNA with quantitative real-time RT-PCR. Thirty-one (65.2%) samples showed SSTR-2 positivity and 6 of nine samples (66.6%) were SSTR-5 positive. Conversely, Ardjomand et al. [22] identified SSTR-2 expression in almost all of the 25 tested UM samples. Furthermore, using ligand competition assay we investigated the binding of radioiodinated RC-160 to membrane fractions of 20 UM specimens. We found that 70% of the human UM samples studied displayed specific SSTRs with a mean K_d_ of 7.14 nM and with a mean B_max_ of 604 fmol/mg membrane protein. It is also important to note that all receptor-positive tumor samples expressed a well-detectable amount of the SSTR-2 or SSTR-5 receptor gene.

Histological distribution of the analyzed samples showed that more than half of the samples (56.1%) were spindle cells, 36.6% were epithelioid, and the remaining 7.3% were mixed histologically. Our findings suggest that the patient’s gender and age are not predicting factors. Our results demonstrate that SSTR-2 and SSTR-5 are expressed in human UM specimens. qRT-PCR results show that SSTR-2 and SSTR-5 are also highly expressed in both UM cell lines investigated. In this study, we provide evidence for the existence of SSTRs in two human UM cell lines and demonstrate that OCM-3 cells express SSTRs at a significantly higher level than OCM-1 cells (*p* < 0.0001). The receptor protein encoded by mRNA for SSTR-2 or SSTR-5 was also demonstrated by western blot in both UM cell lines.

Targeted therapy of cytotoxic peptide analogs consisting of a cytotoxic group, such as a doxorubicin, conjugated to peptide carriers should be more effective and less toxic than conventional systemic chemotherapy. The only major side effect of these analogs is myelosuppression caused by the infrequent chemical cleavage of the cytotoxic radical doxorubicin [57,58]. The high incidence of SST receptors in UM suggests that this type of tumor might be a good candidate for therapy with SST analogs including the targeted cytotoxic peptide AN-162 [24,26]. Since therapy for UM is not effective, our work helps to identify specific molecular targets for the prevention of metastasis or further proliferation of disseminated metastases.

## 4. Materials and Methods

### 4.1. Cell Lines and Culture Conditions

OCM-1 and OCM-3 human primary UM cell lines were kindly provided by the Department of Biophysics and Cell Biology, University of Debrecen, Hungary. In all our in vitro experiments as a positive control CACO-2 (human Caucasian colon adenocarcinoma) cell line was used, which was kindly provided by the Department of Pharmaceutical Technology, University of Debrecen, Hungary. OCM-1 and OCM-3 cell lines were cultured in RPMI 1640 medium supplemented with l-glutamine, 10% fetal bovine serum (FBS), and 1% penicillin/streptomycin in a humidified chamber in 5% CO_2_ at 37 °C. CACO-2 cell line was grown in Dulbecco’s Modified Eagle’s Medium, supplemented with 3.7 g/L NaHCO_3_, 10% (*v*/*v*) heat-inactivated fetal bovine serum (FBS), 1% (*v*/*v*) non-essential amino acids solution, 1% (*v*/*v*) L-glutamine, and 100 IU/mL penicillin/streptomycin in a saturated humidified chamber in an atmosphere of 5% CO_2_ at 37 °C. Cells were subcultured every 3 days using a standard trypsinization procedure. This research received approval from the Institutional Ethics Committee of the University of Debrecen.

### 4.2. Preparation of Uveal Melanoma Tissue Samples from Patients

Specimens of primary human UM were obtained from 46 patients at the time of initial surgical treatment at the Department of Ophthalmology, University of Debrecen, Debrecen, Hungary. The local Institutional Ethics Committee approved the collection and use of these specimens for the current study and informed consent was obtained from these patients. Tumor tissues were immediately frozen in liquid nitrogen and stored at −80 °C until further processing. Histopathological examination of each specimen was undertaken to confirm the presence of cancer with minimal mixed non-malignant tissue. All cancer samples were primary tumors and without metastases. Clinicopathological data of the UM specimens are shown in Table 1.

### 4.3. RNA Isolation and Reverse Transcription PCR

Homogenization of the UM tissue samples were performed with Tissue Ruptor (IKA^®^-WERKE GmbH, Staufen im Breisgau, Germany). Total RNA from tumor tissues and OCM-1 and OCM-3 cells was isolated with NucleoSpin DNA/RNA/Protein Kit (Macherey-Nagel, Düren, Germany) according to manufacturer’s protocol. Quantitative and qualitative assays for RNA were performed using a NanoDrop spectrophotometer (ND-1000, Bioscience, Budapest, Hungary). 

Two hundred fifty nanograms of RNA from each sample were reverse-transcribed into cDNA using Tetro cDNA Synthesis Kit (Bioline Reagents, London, UK). Reaction was performed according to the manufacturer’s instructions. RT-PCR reaction was performed in 25 μL reaction volume with gene specific primers. The primer sequences are shown in Table 2. The reaction consisted of 35 cycles (95 °C for 15 s, 60 °C for 30 s, 72 °C for 10 s) and lasted for 2 min extension at 72 °C. PCR was performed with gene-specific primers for SSTR-1, SSTR-2, SSTR-3, SSTR-4 and SSTR-5 using PCR MyTaq Master Mix (Bioline Reagents). PCR reaction was performed in a C1000 TM Thermal Cycler RT-PCR system (Bio-Rad, Hercules, CA, USA). For SSTR-1, -2, -3, -4, -5 and β-actin an initial denaturing step at 94 °C for 30 s was followed by 30 PCR cycles consisting of denaturation at 94 °C for 15 s, annealing at 60 °C for 30 s and extension at 72 °C for 15 s. β-actin was used as a positive internal control. PCR product was separated in a 1.5% agarose gel containing GelRed and detected in UV light, digitalized with AlphaDigiDoc™ RT (Alpha Innotech, Santa Clara, CA, USA). To determine the size of DNA, 50 bp DNA marker (Bioline Reagents) was used. 

### 4.4. Quantitative Real-Time PCR

Total RNA (1000 ng) was reverse transcribed into cDNA using Tetro cDNA Synthesis Kit (Bioline Reagents) according to the manufacturer’s instruction. To quantity mRNA for SSTR-1, -2, -3, -4 and SSTR-5 real-time RT-PCR method was performed with iTaq™ Universal SYBR^®^ Green Supermix (Bio-Rad) in CFX96 Touch TM Real-Time PCR detection System (Bio-Rad) with a 20 µL final reaction mixture. The reaction was carried out at 95 °C for 10 min, followed by 45 cycles at 95 °C for 15 s and 60 °C for 60 s. β-actin was used as an endogenous reference gene. All real-time amplifications were measured in triplicates. Relative mRNA expression for SSTRs was measured by ∆∆Ct method with threshold cycle times of each target SSTR and β-actin. Template-free and reverse transcription-free controls excluded non-specific amplification and DNA contamination.

### 4.5. Western Blot Analysis

For western blot analysis adherent cells were harvested in M-PER lysis buffer (Thermo Fisher Scientific, Waltham, MA, USA) supplemented with protease inhibitor (Sigma-Aldrich, St. Louis, IL, USA). Total protein concentration was measured by the Pierce BCA protein assay kit (Thermo Fisher Scientific). SDS-PAGE gel electrophoresis was performed under reducing conditions using 10% polyacrylamide gels. Equal amounts of proteins (40 μg) were separated and then transferred to PVDF membrane using wet transfer. After blocking with 5% TBST-milk, the membranes were incubated (overnight, 4 °C) with primary antibodies SSTR-5: anti-somatostatin-receptor-2-rabbit, 1:1000 dilution (ab134152 rabbit monoclonal; Abcam, Cambridge, UK) and anti-somatostatin-receptor-5-rabbit, 1:1000 dilution (ab109495 rabbit monoclonal; Abcam). As a housekeeping gene anti-HPRT was used, 1:1000 dilution (PA-22281 rabbit polyclonal; Cell Signaling Technology, Danvers, MA, USA). After the washing steps, the membrane was incubated with alkaline phosphatase conjugated polyclonal rabbit-anti-mouse secondary antibody, 1:3000 dilution (sc-2771; Santa Cruz Biotechnology Inc., Dallas, TX, USA). Proteins were detected with Alkaline Phosphatase Conjugate Substrate Kit (Bio-Rad).

### 4.6. Radioligand Binding Studies

Radioiodinated derivatives of SST analog RC-160 were prepared using the chloramine-T method and purified using reverse phase high performance liquid chromatography (RP-HPLC) as described earlier [59]. Somatostatin receptor binding studies were performed as previously reported [59] with some minor modifications using in vitro ligand competition assays based on binding of [^125^I]RC-160 as radioligand to uveal melanoma membrane fractions. This radioiodinated ligand was well characterized as reported earlier and demonstrated high affinity binding to SSTR-2 and SSTR-5 [59]. Tumor membrane homogenates were incubated with 50,000–70,000 cpm of radioiodinated RC-160 and 10^−12^–10^−6^ M of nonradioactive peptides as competitors. After 2 h of incubation the binding reactions were terminated, and the bound ligand was separated then counted in a gamma-counter. The LIGAND-PC computerized curve-fitting software of Munson and Rodbard was used to identify the type of receptor binding, dissociation constant (K_d_), and the maximal binding capacity of the receptors (B_max_) [59].

### 4.7. Statistical Analysis

Correlation analysis was carried out among the expression of mRNA for SSTR-1, -2, -3, -4, -5 receptors in OCM-1 and OCM-3 cell lines with the use of GraphPad Prism 7—one way ANOVA test to assess the significance of the expression of SSTR-1–SSTR-5 genes of OCM-1 and OCM-3 cell lines (GraphPad Software Inc., La Jolla, CA, USA).

## 5. Conclusions

Our findings, that a high percentage of human UM samples express SST receptors, support the view that targeted cytotoxic SST analogs such as AN-162 could be used to effectively treat UM. Future investigations are needed to prove the therapeutical and clinical significance of our findings and provide better understanding of the development of human UM. Our studies should help in the early diagnosis of metastasis and in the use of SST analogs in subsequent improved targeted therapies.

## Figures and Tables

**Table 1:** Clinicopathological characteristics and SSTR results of uveal melanoma specimens.

Number of Case	Age (Years)	Sex	Histology	mRNA for SSTR-2	mRNA for SSTR-5	SSTR Binding	
						K_d_ (nM)	B_max_ (fmol/mg Protein)
1	30	female	Spindle	−	N/A	N/A	N/A
2	33	female	Spindle	+	N/A	N/A	N/A
3	35	male	Spindle	−	N/A	N/A	N/A
4	38	male	Spindle	+	N/A	6.42	304
5	39	male	N/A	+	N/A	N/A	N/A
6	39	male	Epithelioid	−	+	9.19	527
7	43	male	Spindle	+	N/A	N/A	N/A
8	45	male	Spindle and Epithelioid	+	N/A	N/A	N/A
9	47	male	Epithelioid	−	−	−	−
10	47	male	Epithelioid	+	+	8.53	1052
11	50	female	Spindle	+	N/A	N/A	N/A
12	50	female	Spindle	+	N/A	N/A	N/A
13	52	male	Spindle	+	N/A	N/A	N/A
14	53	male	Epithelioid	+	N/A	6.24	260
15	53	male	N/A	−	N/A	N/A	N/A
17	60	male	Epithelioid	+	N/A	N/A	N/A
18	61	male	Spindle	+	N/A	N/A	N/A
19	62	female	Epithelioid	+	N/A	5.58	583
20	64	male	N/A	+	N/A	N/A	N/A
21	64	male	Spindle	−	+	4.75	482
22	65	male	Spindle	−	+	3.18	467
23	66	male	Epithelioid	+	N/A	N/A	N/A
24	67	male	Epithelioid	+	N/A	5.04	509
25	68	female	Spindle	−	N/A	−	−
26	68	male	Spindle	+	N/A	N/A	N/A
27	68	female	Epithelioid	+	N/A	N/A	N/A
28	69	male	Spindle and Epithelioid	+	N/A	9.48	774
29	70	male	Spindle and Epithelioid	+	N/A	N/A	N/A
30	70	male	Spindle	+	N/A	4.37	985
31	70	male	Spindle	−	−	−	−
32	72	female	Epithelioid	+	N/A	N/A	N/A
33	72	male	Epithelioid	+	N/A	11.8	473
34	72	male	Epithelioid	−	N/A	N/A	N/A
35	75	female	Spindle	−	N/A	−	−
36	75	female	Epithelioid	+	N/A	N/A	N/A
37	75	female	Spindle	+	N/A	11.1	668
38	75	female	Spindle	−	+	3.57	392
39	76	male	Spindle	−	N/A	−	−
40	76	male	Spindle	+	N/A	N/A	N/A
41	76	male	Spindle	−	+	10.7	980
42	79	female	Epithelioid	+	N/A	N/A	N/A
43	79	female	N/A	+	N/A	N/A	N/A
44	79	female	N/A	+	N/A	N/A	N/A
45	80	female	Epithelioid	−	−	−	−
46	84	female	Spindle	+	N/A	N/A	N/A

Abbrevations: N/A: not analyzed. “−”: negative. “+”: expression was observed. K_d_: dissociation constant. B_max_: maximal binding capacity.

**Table 2:** The sequences of SSTR-1, -2, -3, -4, -5 and β-actin primers used for qRT-PCR assay.

Target	Forward Sequence	Reverse Sequence
SSTR-1	tgagtcagctgtcggtcatc	ggaaagagcgcttgaagttg
SSTR-2	ctttgtggtggtcctcacct	gcagaggacattctggaagc
SSTR-3	ttcctctcctaccgcttcaa	ctcctcctcatcctcctcct
SSTR-4	tctttgtgctctgctggatg	ggataagggacacgtggttg
SSTR-5	tctttgtgctctgctggatg	gttggcgtaggagaggatga
β-actin	ggcatcctcaccctgaagta	ggggtgttgaaggtctcaaa
